# PCSK9, a novel immune and ferroptosis related gene in abdominal aortic aneurysm neck

**DOI:** 10.1038/s41598-023-33287-9

**Published:** 2023-04-13

**Authors:** Junli Zhuang, Hua Zhu, Ziqi Cheng, Xinyao Hu, Xiaohui Yu, Jie Li, Huagang Liu, Peng Tang, Ying Zhang, Xiaoxing Xiong, Hongping Deng

**Affiliations:** 1 Department of Vascular Surgery, Renmin Hospital of Wuhan University, NO. 99 Zhang Zhidong Road, Wuchang District, Wuhan, 430060 China; 2 Department of Neurosurgery, The Affiliated Huzhou Hospital, Zhejiang University School of Medicine (Huzhou Central Hospital), NO. 1558 North Sanhuan Road, Huzhou, 313003 Zhejiang China; 3 Department of Neurosurgery, Renmin Hospital of Wuhan University, NO. 99 Zhang Zhidong Road, Wuchang District, Wuhan, 430060 China; 4 Cancer Center, Renmin Hospital of Wuhan University, NO. 99 Zhang Zhidong Road, Wuchang District, Wuhan, 430060 China

**Keywords:** Bioinformatics, Biomarkers

## Abstract

The gene expression profile of abdominal aortic aneurysm (AAA) neck is not fully understood. The etiology of AAA is considered to be related to atherosclerosis and the inflammatory response, involving congenital, genetic, metabolic, and other factors. The level of proprotein convertase subtilisin/kexin type 9 (PCSK9) is related to those of cholesterol, oxidized low-density lipoprotein, and triglycerides. PCSK9 inhibitors have significant effects on lowering LDL-cholesterol, reversing atherosclerotic plaques, and reducing the risk of cardiovascular events and have been approved by several lipid-lowering guidelines. This work was aimed to investigate the potential role of PCSK9 in the neck of AAA. We extracted the expression dataset (GSE47472) containing 14 AAA patients and 8 donors and single-cell RNAseq (scRNA-seq) data (GSE164678) of CaCl_2_-induced (AAA) samples from the Gene Expression Omnibus dataset. Through bioinformatics methods, we found that PCSK9 was up-regulated in the proximal neck of human AAA. In AAA, PCSK9 was mainly expressed in fibroblasts. Additionally, immune check-point PDCD1LG2 was also expressed higher in AAA neck than donor, while CTLA4, PDCD1, and SIGLEC15 were down-regulated in AAA neck. The expression of PCSK was correlated with PDCD1LG2, LAG3, and CTLA4 in AAA neck. Additionally, some ferroptosis-related genes were also down-regulated in AAA neck. PCSK9 was also correlated with ferroptosis-related genes in AAA neck. In conclusion, PCSK9 was highly expressed in AAA neck, and may exert its role through interacting with immune check-points and ferroptosis-related genes.

## Introduction

Abdominal aortic aneurysm (AAA) is one of the arterial degenerative diseases, with an incidence rate of 5–10% in the elderly. With age, the incidence of AAA, the rate of vascular expansion, and the risk of vascular rupture gradually increases. Once an AAA ruptures, the mortality rate can be as high as 90%^1^. The most commonly used treatment options for AAA include classic open repair (OR) and AAA endovascular exclusion (EVAR). Open surgical repair is convenient and has positive mid- and long-term effects, but it is traumatic and is susceptible to complications. Although EVAR has the advantages of being minimally invasive, safe with fewer complications, and enabling a rapid recovery, the treatment plan is expensive and has a significant impact on the aneurysm shape^1,2^. Cross-section of the neck of the AAA may have an effect on stent deployment^3^. It has been reported that several risk factors, including a short or highly angulated proximal aortic neck, are associated with long-term outcomes after endovascular or open AAA repair^4^. However, the gene profile of AAA neck remains to be unknown. Therefore, exploring the mechanism of occurrence and development of AAA neck, finding suitable targets based on the mechanisms of action, and screening for and designing targeted drugs that intervene and control the progress of AAA neck have extremely significant clinical value.

Proprotein convertase subtilisin/kexin type 9 (PCSK9) is the ninth member of the subtilisin family of proprotein convertases. The human PCSK9 gene is located at chromosome 1p3213, is about 22 kb in length, contains 12 exons, and encodes 692 amino acids. PCSK9 is mainly produced by the liver. It can bind to the low-density lipoprotein receptor (LDL-R) on liver cells to reduce the liver’s ability to remove LDL-cholesterol. Therefore, PCSK9 is an important target of lipid-lowering drugs. High levels of PCSK9 expression in the nervous system promote inflammation^5^, which is closely associated with the occurrence of AAA. Additionally, a previous study that verified the high PCSK9 intensity in aneurysm^6^. PCSK9 has been reported to directly interface with, and decrease, endogenous cell surface CD36, thus decreasing the internalization of fatty acids in adipocytes. It has also been demonstrated that PCSK9 expressed around calcification areas formed by vascular smooth muscle cells, especially those of the synthetic phenotype, was associated with the development of acute aortic dissection, suggesting that PCSK9 is associated with the loss of structural integrity of the aorta and subsequent imbalanced vasoconstriction^7^. In addition, infection of C57BL/6 mice with AAV that obtained stable expression of functionally mutated mouse PCSK9 provided a model for rapid enhancement of AngII-induced AAA^8^. These researches demonstrate that PCSK9 may play a role in the development of AAA, but the exact mechanism of action of PCSK9 has not been reported, which is the reason why we have pursued this gene.

Inhibition of PCSK9 has been reported to enhance the effectiveness of immune checkpoint therapy for cancer^9,10^. Immune checkpoint also paly important role in the abdominal aortic aneurysm progression, for example, immune checkpoint PD-1 mediates abdominal aortic aneurysm and pseudoaneurysm progression^11^. However, the latent function of PCSK9 with immune checkpoint in AAA neck has not been investigated. Previous research has shown that iron levels in the AAA aortic tissue are significantly increased and that the oxidative stress and inflammatory response caused by iron overload aggravate the development of AAA^12^. Ferroptosis may influence the AAA progression by modulating vascular smooth muscle cells^13^, which are the primary cells that construct the medial layer of aorta. It also has been reported that ferroptosis exerts a crucial action in AAA^14^. However, whether PCSK9 have a correlation with ferroptosis has not been studied.

In this work, we investigated the expression of PCSK9 in donor and AAA neck and its association with immune checkpoints or ferroptosis-related genes mainly through bioinformatics. We found that PCKS9 was increased in AAA neck tissues and may correlated with immune checkpoints or ferroptosis.

## Materials and methods

### Data acquisition and pre-processing

The gene expression data (GSE47472) of proximal neck of human abdominal aortic aneurysm was obtained from the Gene Expression Omnibus (GEO) (http://www.ncbi.nlm.nih.gov/geo/). In total, 22 AAA neck biopsies were obtained, including 14 AAA neck samples from patients who underwent open AAA repair and 8 normal samples from heart organ donors after brain death. The data of GSE47472 in GEO were downloaded as MINiML formatted file format and normalized by log2 transformation. The normalize quantiles function of the preprocessCore package^15^ were used to normalize the microarray data using R software (version 4.0.3). Additionally, the scRNA-seq data (GSE164678) containing 1 AAA and 1 sham sample was obtained from the GEO database. All methods were carried out in accordance with relevant guidelines and regulations.

### Different expression genes analysis

Limma package (version: 3.40.2)^16^ of R software (version: 4.0.3) was employed to study the different expression genes (DEGs). We evaluated the adjusted *p*-value to correct for false positive results in GEO dataset. “Adjusted *p* < 0.05 and |Log FC|> 1” were used as the thresholds to screen for the DEGs. The heat map was displayed by the R package ‘pheatmap’ (https://rdocumentation.org/packages/pheatmap/versions/1.0.8) by using R software (version 4.0.3). The student t-test was used for the PCSK9 expression analysis in normal (n = 8) and AAA neck group (n = 14). The box plot of PCSK9 expression analysis was performed by the R package ggplot2.

### ScRNAseq data analysis

We further downloaded the scRNAseq dataset (GSE164678) of 1 AAA and 1 sham sample of mouse model from GEO database to locate the PCSK9 expression. The following screening criteria were used for scRNAseq data: genes detected in > 3 cells, percentage of mitochondrial genes < 10% and cells with > 200 distinct genes. We integrated the AAA and sham data using CAA algorithm of Seurat package (version 4.0.2)^17^. Then, data normalization and scaling were carried out and then followed by dimension reduction at 3 stages of analysis including the selection of variable genes, principal component analysis, and uniform manifold approximation and projection (UMAP). To make sure that the known major aortic cell types were separated without excessively subclustering, cell clustering was evaluated at a range of predetermined resolution scales (resolution: 0.5). The expression levels of cell‐specific markers from CellMarker database^18^ were applied to identify the cell types as a previous study reported^19^. Then, PCSK9 expressions in each cell population were evaluated.

### Animals and AAA model

12-week-old wild-type C57BL/6J mice (n = 10) obtained from the Laboratory Animal Center of China Three Gorges University (Hubei, China) were raised in an SPF environment in the Animal Experiment Center of Renmin Hospital of Wuhan University. The mice were adaptively fed for a week before the experiment. All experimental protocols in this work were followed the local animal ethics procedures and approved by the ethics committee of Renmin Hospital of Wuhan University and carried out in accordance with ARRIVE guidelines. Ten mice were randomly divided into two group: sham (n = 5) and AAA model (n = 5). Mice were anesthetized with 3% isoflurane gas and maintained under anesthesia with oxygen containing 1.5% isoflurane (oxygen flow rate of 0.3 L/min) delivered by mask and then operated under aseptic conditions^20^. The subrenal abdominal aorta was exposed and separated from the surrounding retroperitoneal tissue. The morphology of the abdominal aorta was recorded microscopically, and the maximum diameter of the abdominal aorta was measured; CaCl_2_ (0.5 mol/L) was applied to the external surface of the aorta for 15 min using cotton gauze strips. For sham group, CaCl_2_ solution was replaced with 0.9% NaCl solution. The aorta was then flushed 3 times with sterile saline. After 6 weeks of model establishment, euthanasia was performed and the neck tissue of abdominal aortic aneurysm was removed.

### Real-time qPCR

The mice (n = 5/group) were deeply anesthetized and euthanized with an overdose of isoflurane, then the abdominal aortic aneurysm neck tissues were removed. The total RNA was extracted from the AAA neck with TRIzol (TaKaRa, Shiga, Japan) as our previous study^21^. A PrimeScript RT kit (Takara, RR047A, Japan) was then used to perform reverse transcription to reverse transcribe the RNA into cDNA. Real-time qPCR in duplicate was performed using SYBR-Green PCR Mix (TaKaRa, RR820A, Japan) and an ABI PRISM 7500 thermal cycler (Applied Biosystems, CA). The amplification primers was designed and synthesized by BGI Genomics (Shenzhen, China) as: β-actin: F:GGCTGTATTCCCCTCCATCG, R: CCAGTTGGTAACAATGCCATGT; PCSK9: F: GAGACCCAGAGGCTACAGATT, R: AATGTACTCCACATGGGGCAA. Relative PCSK9 mRNA expression was measured using β-actin as a control. The 2^−ΔΔCt^ method was used to estimate the relative expression of PCSK9.

### Immune checkpoints analysis

We extracted the expression value of 8 immune checkpoint genes in the AAA neck (n = 14) and donor group (n = 8). The differential expression of immune checkpoint genes between the normal and AAA neck groups were investigated using Wilcoxon test. The box plot was conducted by the R package ggplot2^22^ using R software (version: 4.0.3); the heat map was implemented by the R package pheatmap. Spearman’s correlation analysis was applied to describe the correlation between PCSK9 and immune checkpoint genes.

### Ferroptosis-related genes analysis

Twenty-four ferroptosis-related genes were obtained from the systematic analysis of the aberrances and functional significance of ferroptosis in cancer by Liu et al.^23^. The ferroptosis-related genes’ expression data were extracted in 8 donors and 14 AAA patients. The differential expression of ferroptosis-related genes between the normal and AAA neck groups were tested using Wilcoxon test. We visualized the expression of these genes in AAA neck and normal tissues by the R package ggplot2. We employed Spearman's correlation analysis to evaluate the correlation between PCSK9 and ferroptosis-related genes.

### Protein–protein interaction (PPI) network of PCSK9

STRING database (https://cn.string-db.org/) is an objective, extensive global network aimed at collecting, integrating and scoring the published protein–protein interaction (PPI) information, and at supplementing this data through scientific calculations and predictions^24^. To further investigate the potential proteins that interact with PCSK9, we employed the dataset STRING for PPI network associated with PCSK9. The confidence set in the STRING database as: minimum required interaction score: medium confidence (0.400).

GeneMANIA (https://genemania.org/) database is a flexible and user-friendly webtool designed to formulate gene function hypotheses, prioritize genes for functional analysis, and generate analysis gene list^25^. It can discover and predict proteins with similar functions based on large amounts of genomic and proteomic data. This webtool was also applied for further investigation of the PPI network of PCSK9.

### Prediction of miRNA-target gene regulatory relationships and transcription factors (TFs)

To predict the miRNAs that target PCSK9, we used the Enrichr software (http://amp.pharm.mssm.edu/Enrichr/) (*p* < 0.05). To explore whether TFs are involved in the dysregulation of PCSK9, we used the Signaling Pathways Project (http://www.signalingpathways.org/) to predict TFs for PCSK9. We selected “human” for the biosample category with other default options. Additionally, the TFs were also analyzed with Enrichr software based on the TRANSFAC and JASPAR databases (*p* < 0.05)^26^. Cytoscape (version 3.9.1) was used for the construction to integrate the TF-target and miRNA-target networks.

### Function enrichment analysis

We firstly analyzed the correlation of PCSK9 with other genes in AAA neck by Spearman’s correlation analysis corrected by Benjamini–Hochberg. We next performed the Gene ontology (GO) and Kyoto Encyclopedia of Genes and Genomes (KEGG) enrichment analysis by Gene Set Enrichment Analysis (GSEA) software (version 4.2.3). GSEA analysis is an algorithm to evaluate the variability of gene sets between two different phenotypes^27^ and KEGG is a knowledge base for systematic analysis of functions of genes^28^. The normalized GSE47472 dataset as input RNA-seq data and used PCSK9 expression as the phenotypic label (*p* < 0.05). The reference gene sets were c5.go.v2022.1.Hs.symbols.gmt and c2.cp.kegg.v2023.1.Hs.symbols.gmt. Then, the results were integrated in a bubble diagram based on the normalized enrichment score (NES) value.

### Statistical analysis

GraphPad Prism software 8.0.2 was employed for statistical analysis. Results were showed in a form of mean ± SD. Student t-test or wilcoxon test was adopted for statistical comparison between control (donor) and AAA neck. Spearman’s correlation analysis was used to evaluate the correlation between PCSK9 and ferroptosis- and immune checkpoint-related genes. *p* < 0.05 was regarded as statistical significance.

## Results

### DEGs and PCSK9 expression in AAA neck

**Figure 1:**
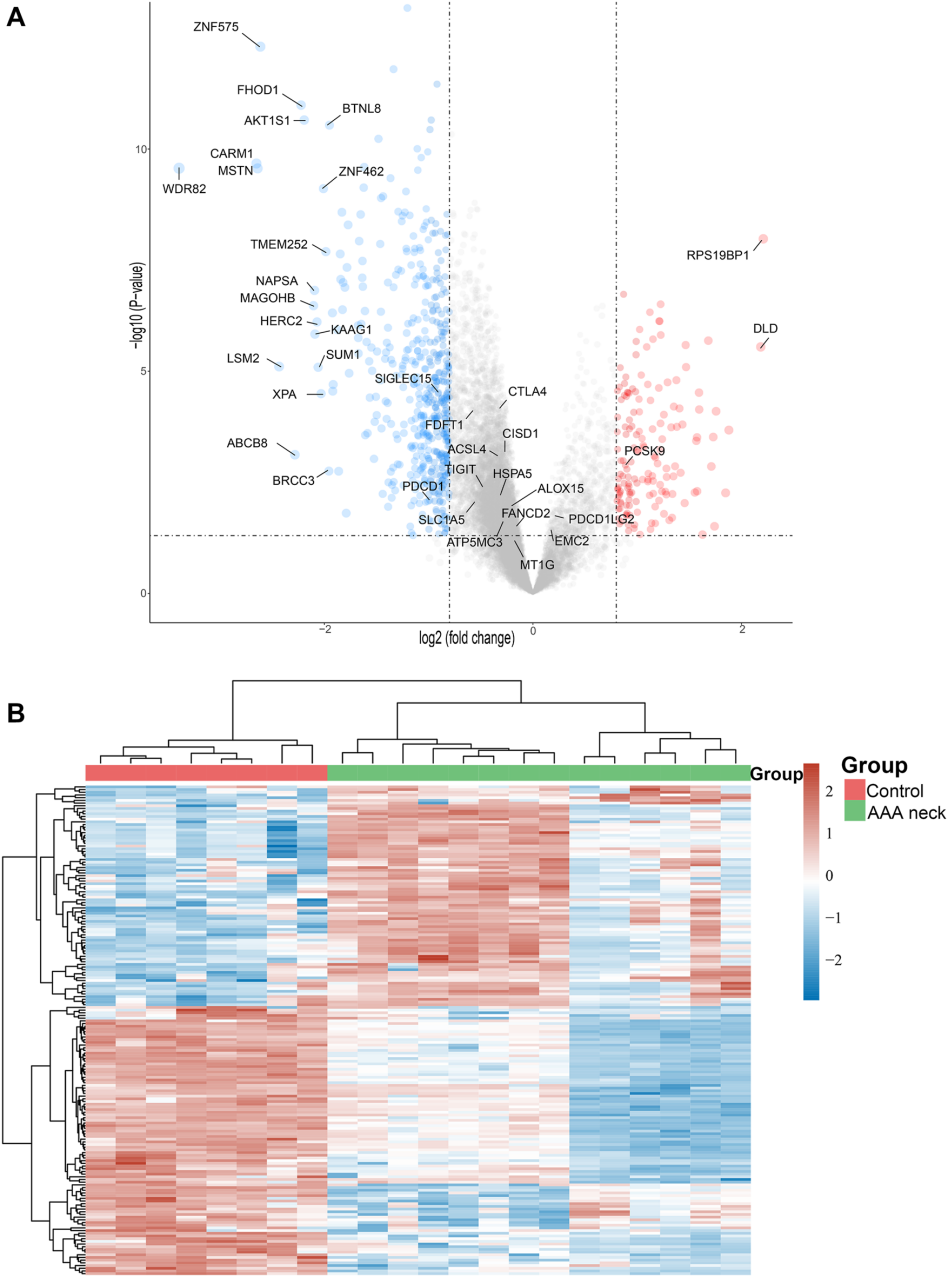
Different expression genes in AAA neck compared to normal group. (**A**) Volcano plot showing the up- and down-regulated genes in AAA neck (n = 14) compared with normal tissues (n = 8) from GSE47472 dataset in GEO (Adjusted *p* < 0.05 and |Log FC|> 1). Red dots represent up-regulation. Blue dots represent down-regulation. (**B**) Through using R software (version: 4.0.3) and R package ‘pheatmap’ (version 1.0.8), heat map was constructed to show the top 50 up- and down-regulated genes in AAA neck (n = 14) than donors (n = 8). Red represents up-regulation. Blue represents down-regulation.

**Figure 2:**
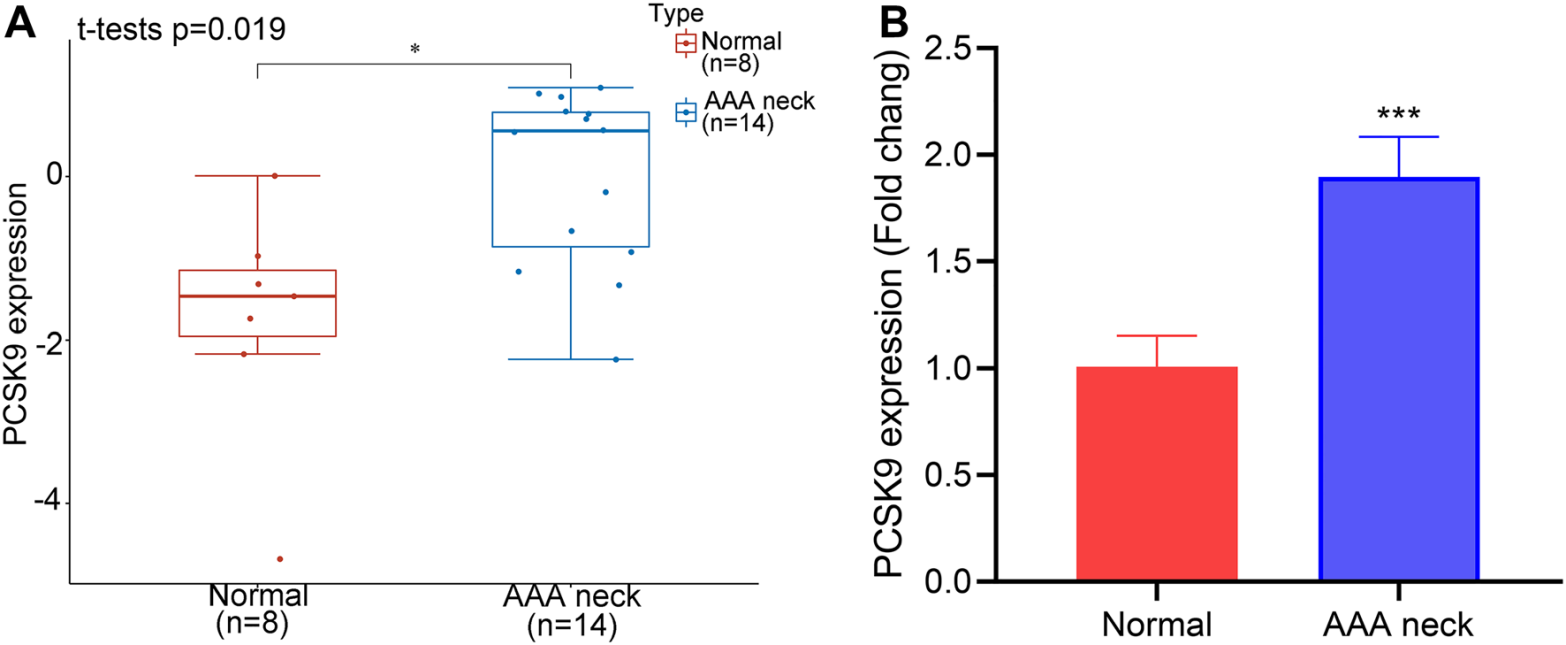
The expression of PCSK9 in AAA neck and normal tissues. (**A**) The expression of PCSK9 in human AAA neck (n = 14) and normal tissues (n = 8) in GSE47472 dataset from GEO database. Student t-test was used to compare the different expressions. *p < 0.05. (**B**) The RT-qPCR results showed the PCSK9 mRNA expression in AAA neck of AAA mice model (n = 5) and normal tissues (n = 5). Student t-test was used to compare the mRNA expressions of PCSK9. ***p < 0.001.

A total of 330 DEGs were identified with the threshold at “adjusted *p* < 0.05 and |Log FC|> 1. The DEGs contained 248 down-regulated genes and 82 up-regulated genes. Several up-regulated or downregulated genes were visualized in Fig. 1A. DLD and RPS19BP1 were down-regulated and the up-regulated genes contained ZNF575, FHOD1, AKT1S1, CARM1, BTNL8, TMEM252, BRCC3, LSM2, NAPSA, etc. The 50 up-regulated and 50 down-regulated genes with the greatest difference were shown in Fig. 1B. PCSK9 was up-regulated in AAA neck than in normal tissues (p = 0.019) (Fig. 2A). Our RT-qPCR results confirmed the overexpression of PCSK9 (*p* < 0.001) (Fig. 2B).

### Cellular localization of the PCSK9 in AAA

**Figure 3:**
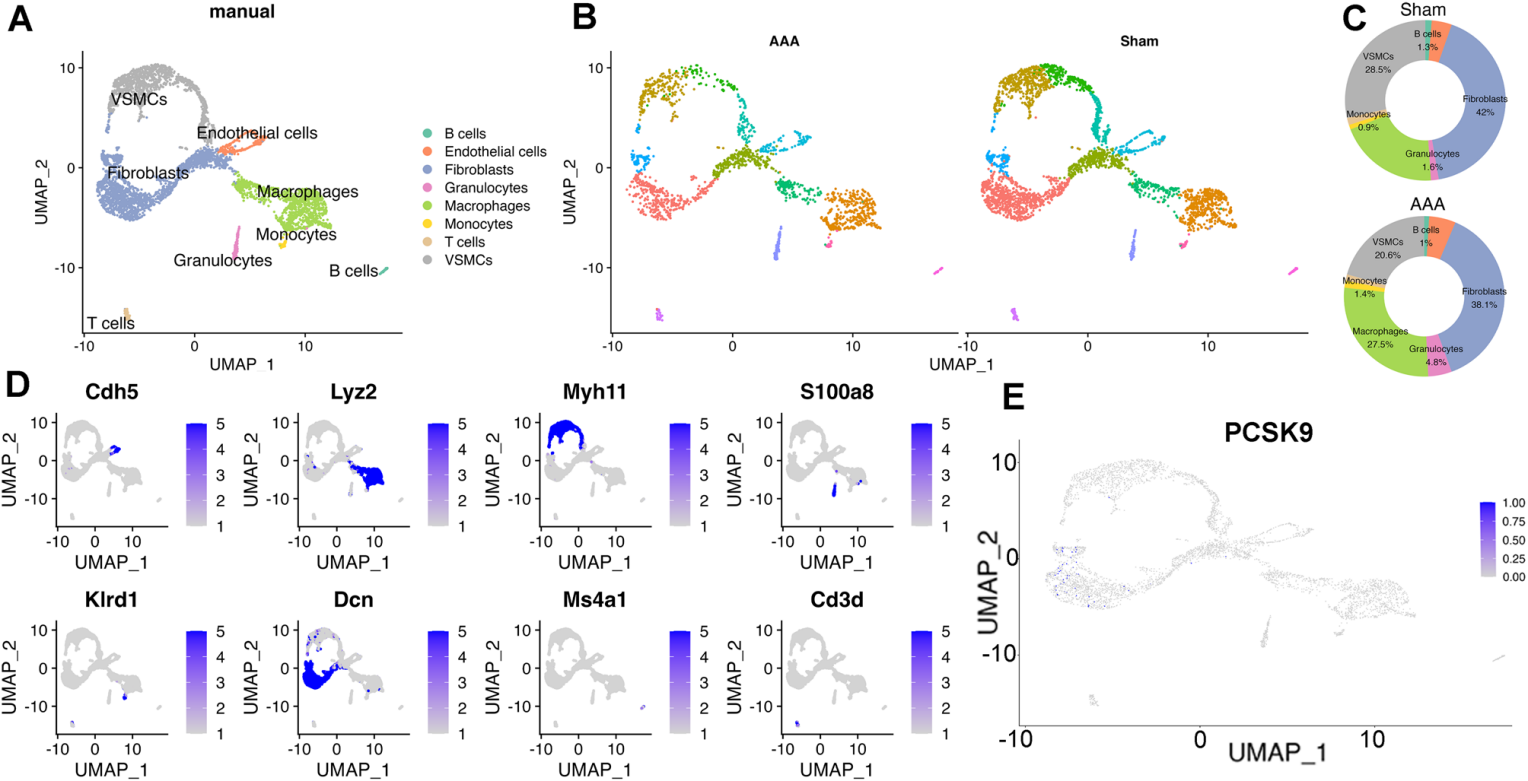
Identification of the cell composition changes in AAA and cellular localization of PCSK9 by scRNA-seq data (GSE164678) analysis. (**A**) Separate eight cell clusters according to uniform manifold approximation and projection (UMAP) plot. (**B**) Cell distributions in AAA and sham samples are shown in UAMP. (**C**) Pie plot showing the percentages of each cell population in AAA and sham groups. (**D**) Top marker genes of these eight cell clusters. (**E**) UMAP plots showing that PCSK9 was mainly expressed in fibroblasts in AAA.

We further revealed the aortic cellular localization of PCSK9 in AAA by scRNA-seq data (GSE164678) analysis. After integrating the AAA and sham data, unbiased cell clustering analysis identified 8 cell lineages including B cells, endothelial cells (ECs), fibroblasts, VSMCs, granulocytes, macrophages, T cells, and monocytes (Fig. 3A). We evaluated the changes of cell composition and found that the VSMCs (AAA vs. Sham: 20.6% vs. 28.5%) and fibroblasts (AAA vs. Sham: 38.1% vs. 42%) were decreased in AAA compared with sham group, while the macrophages and granulocytes populations were increased (Fig. 3B,C). The top marker genes of these eight cell clusters were exhibited in Fig. 3D. Subsequently, we analyzed the cellular localization of PCSK9 expression in AAA, and the results showed that PCSK9 was mainly expressed in fibroblasts (Fig. 3E).

### The expression of immune checkpoints in AAA neck

**Figure 4:**
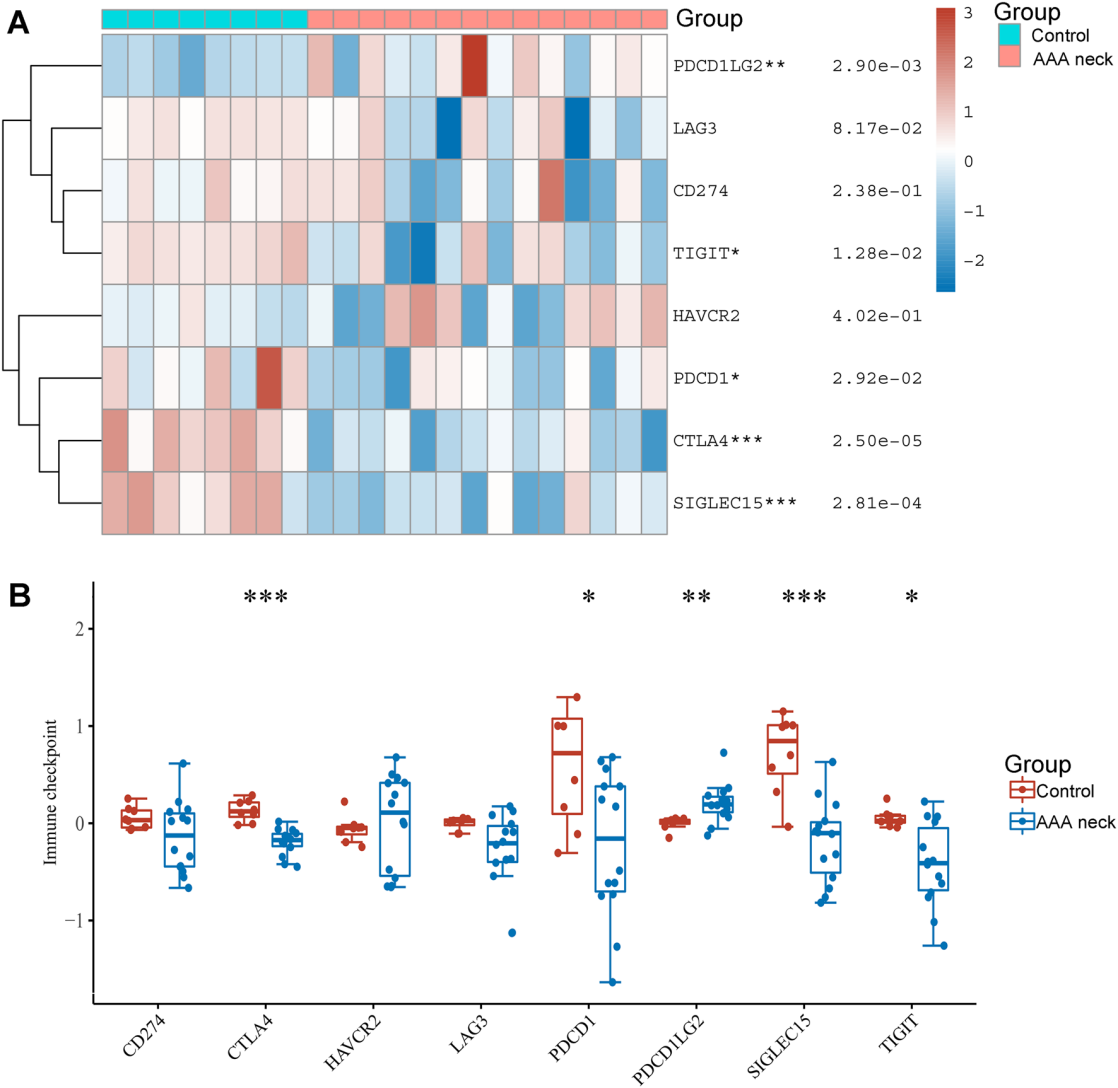
The expression of immune checkpoints in AAA neck and normal tissues. (**A**) Through using R software (version: 4.0.3) and R package ‘pheatmap’ (version 1.0.8), heat map was established to show the expression of immune checkpoints in AAA neck (n = 14) and normal tissues (n = 8) in GSE47472 dataset from GEO database. Red represents up-regulation. Blue represents down-regulation. (**B**) Box plots showing the expression of immune checkpoints in AAA neck (n = 14) and normal tissues (n = 8) in GSE47472 dataset from GEO database. Wilcoxon test was employed to compare the differential expression of immune checkpoints. *p < 0.05, **p < 0.01, ***p < 0.001 AAA neck *vs.* control group.

It has been reported that immune checkpoint paly important role in the abdominal aortic aneurysm progression, for example, immune checkpoint PD-1 mediates abdominal aortic aneurysm and pseudoaneurysm progression. Herein, we investigated the expression levels of PCSK9 in AAA neck and normal group. However, the expression of immune checkpoints in AAA neck has not been studied. We found that immune checkpoints PDCD1LG2 (PD-L2) was at higher expression levels in AAA neck than in normal group (*p* = 2.90e−03) (Fig. 4A,B). However, the expression of TIGIT (*p* = 1.28e−02), PDCD1 (*p* = 2.92e−02), CTLA4 (*p* = 2.50e−05), and SIGLEC15 (*p* = 2.81e−04) were down-regulated in AAA neck (Fig. 4A,B). These results indicated that immune checkpoints CTLA4, PDCD1, PDCD1LG2 (PD-L2), SIGLEC15 and TIGIT may be involved in the progression of AAA neck.

### The correlation between PCSK9 and immune checkpoints in AAA neck

**Figure 5:**
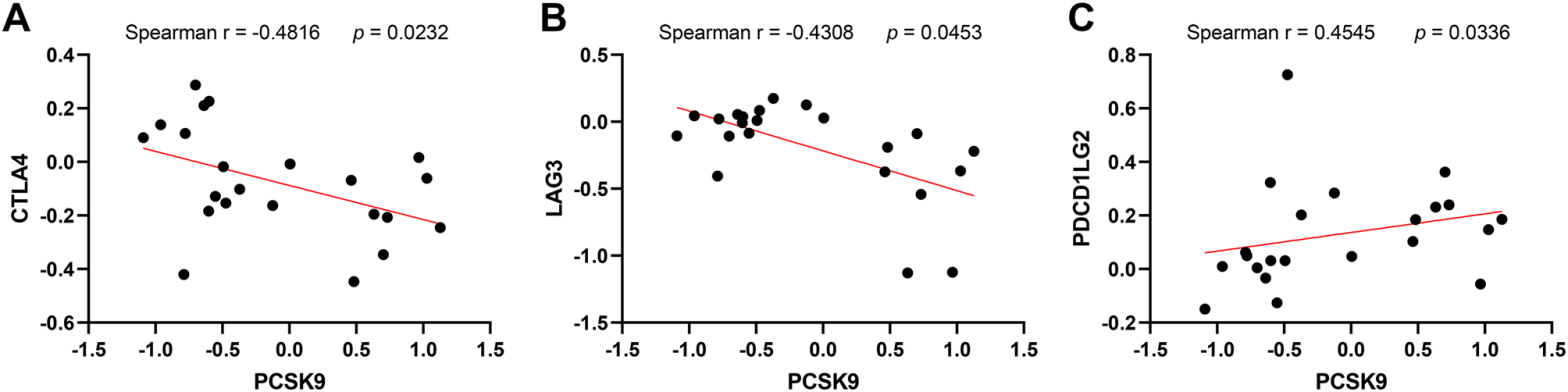
The correlation between PCSK9 and immune checkpoints in AAA neck and donor tissues. (**A**) Spearman’s correlation analysis of the expression data from GSE47472 showed that PCSK9 was negatively related to CTLA4. Spearman’s Rank Correlation Coefficient (Spearman r =  − 0.4816, *p* = 0.0232). (**B**) Spearman’s correlation analysis of the expression data from GSE47472 showed PCSK9 was negatively related to LAG3 (Spearman r =  − 0.4308, *p* = 0.0453). (**C**) Spearman’s correlation analysis of the expression data from GSE47472 showed PCSK9 was positively related to PDCD1LG2 (Spearman r =  − 0.4308, *p* = 0.0453).

To further investigate the potential interaction between PCSK9 and immune checkpoints in AAA neck. We performed spearman’s correlation analysis between PCSK9 and these immune checkpoints. PCSK9 was negatively associated with CTLA4 (r =  − 0.4816, *p* = 0.0232) (Fig. 5A) and LAG3 (r =  − 0.4308, *p* = 0.0453) (Fig. 5B), while positively correlated to PDCD1LG2 (PD-L2) (r =  − 0.4545, *p* = 0.0336) (Fig. 5C).

### The expression of ferroptosis-related genes in the neck of AAA

**Figure 6:**
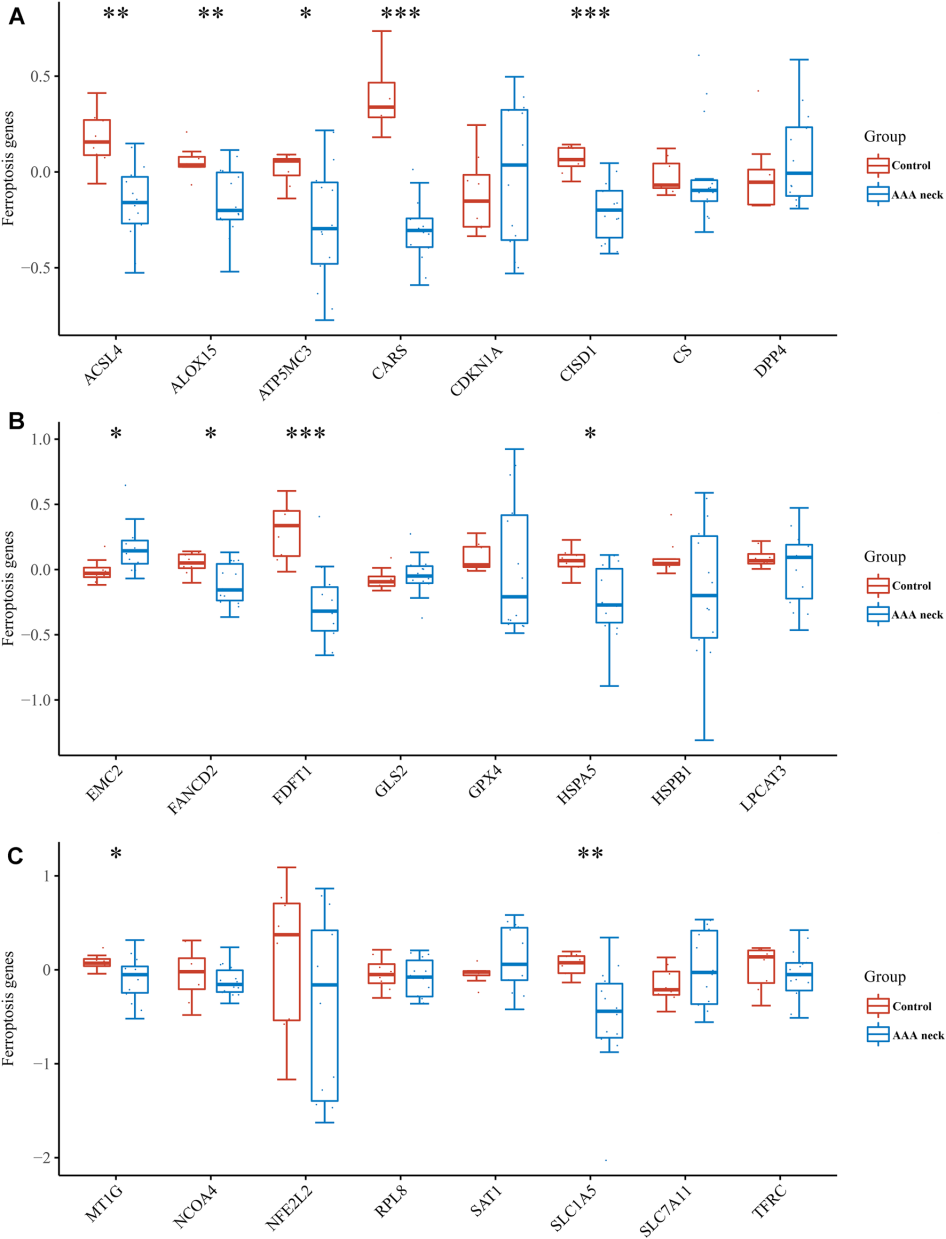
The expression of 24 ferroptosis related genes in the neck of AAA and normal tissues. (**A–C**) The box plots showing the expression of ferroptosis related genes in human AAA neck (n = 14) and donor tissues (n = 8) in GSE47472 dataset. Wilcoxon test was used to compare the differential expression of ferroptosis related genes. *p < 0.05, **p < 0.01, ***p < 0.001 AAA neck *vs.* control group.

Thereafter, we evaluated the expression of 24 ferroptosis-related genes in AAA neck. As presented in Fig. 6A–C, the expression of ACSL4, ALOX15, ATP5MC3, CARS, CISD1, FANCD2, FDFT1, HSPA5, MT1G, and SLC1A5 were down-regulated in AAA neck than in donors. However, the expression of EMC2 was up-regulated in AAA neck (Fig. 6B). These ferroptosis-related genes may exert potential action in the pathogenesis of AAA neck.

### The correlation between PCSK9 and ferroptosis-related genes in AAA neck

**Figure 7:**
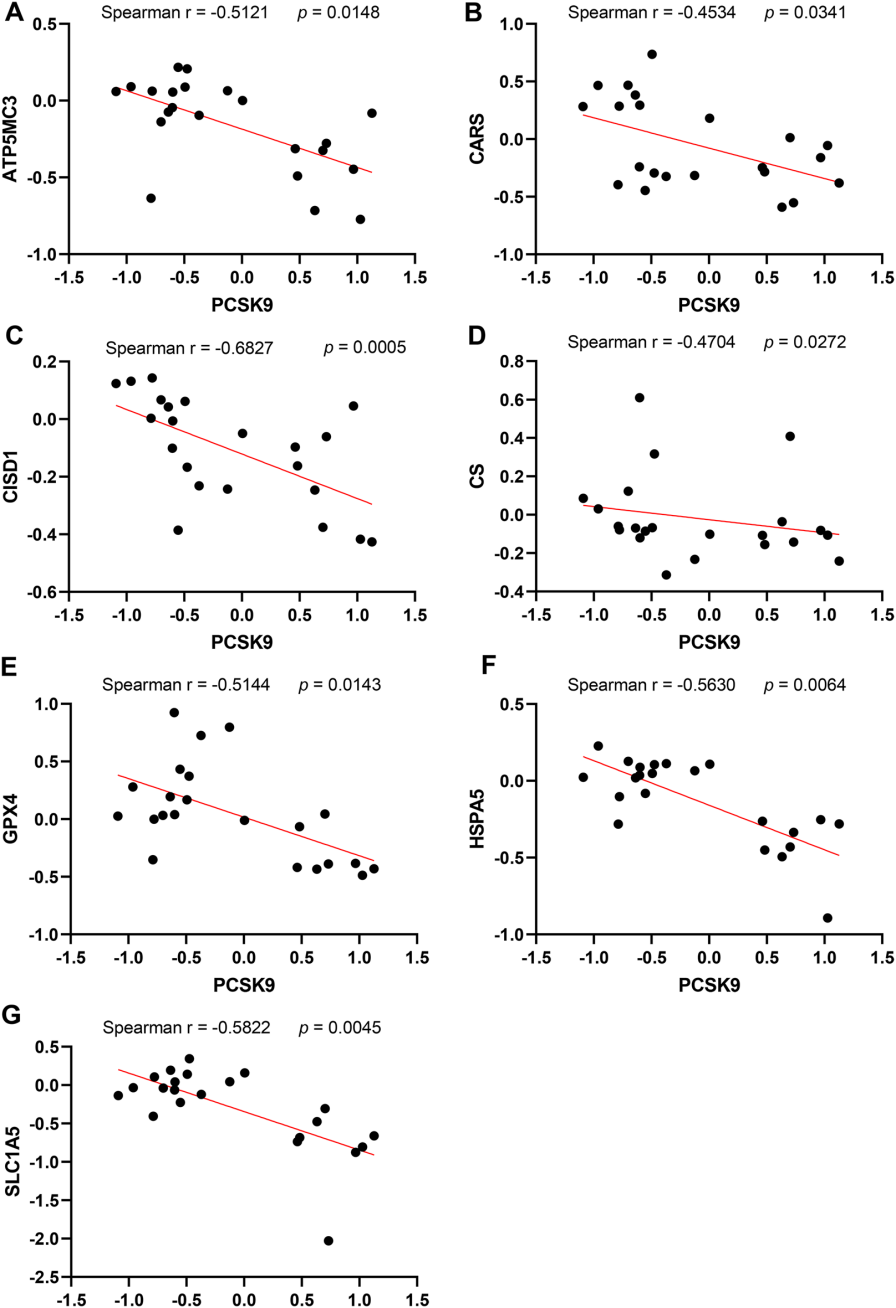
The correlation between PCSK9 and ferroptosis related genes in human AAA neck and normal tissues. Spearman’s correlation analysis of the expression data from GSE47472 from GEO database showed that PCSK9 was negatively related to ATP5MC3 (Spearman r =  − 0.5121, *p* = 0.0148) (**A**), CARS (Spearman r =  − 0.4534, *p* = 0.0341) (**B**), CISD1 (Spearman r =  − 0.6827, *p* = 0.0005) (**C**), CS (Spearman r =  − 0.4704, *p* = 0.0272) (**D**), GPX4 (Spearman r =  − 0.5144, *p* = 0.0143) (**E**), HSPA5 (Spearman r =  − 0.5630, *p* = 0.0064) (**F**), and SLC1A5 (Spearman r =  − 0.5822, *p* = 0.0045) (**G**).

Similarly, we investigated the relationship between PCSK9 and these ferroptosis-related genes. We discovered that PCSK9 was negatively related to some ferroptosis-related genes, including ATP5MC3 (r =  − 0.5121, *p* = 0.0148) (Fig. 7A), CARS (r =  − 0.4534, *p* = 0.0341) (Fig. 7B), CISD1 (r =  − 0.6827, *p* = 0.0005) (Fig. 7C), CS (r =  − 0.4704, *p* = 0.0272) (Fig. 7D), GPX4 (r =  − 0.5144, *p* = 0.0143) (Fig. 7E), HSPA5 (r =  − 0.5630, *p* = 0.0064) (Fig. 7F), and SLC1A5 (r =  − 0.5822, *p* = 0.0045) (Fig. 7G). These results illustrated that PCSK9 may be involved in the formation of AAA neck though the regulation of ferroptosis.

### Potential protein, miRNA, and TFs regulating PCSK9

**Figure 8:**
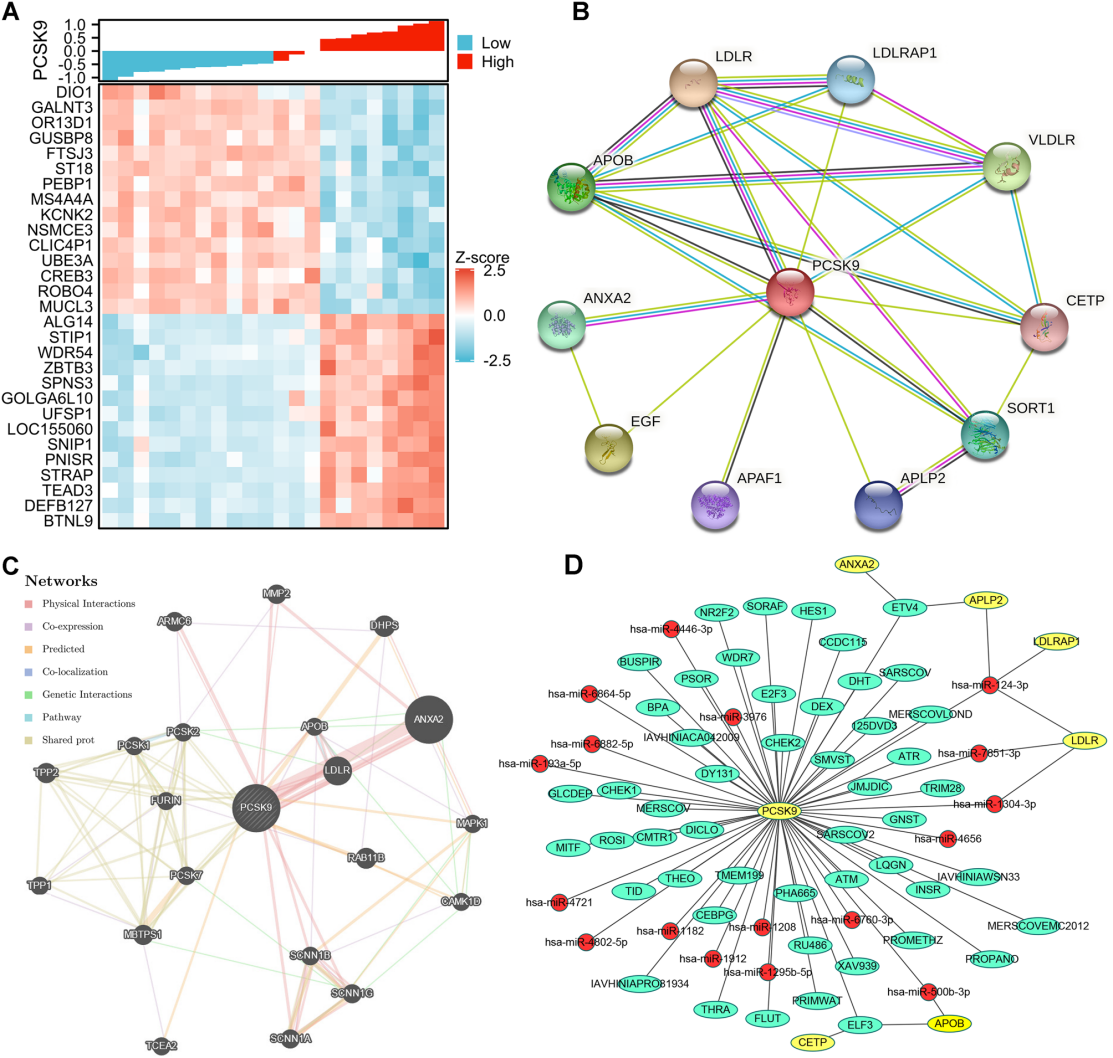
Potential protein, miRNA, and TFs regulating PCSK9. (**A**) Top 15 genes positive and negative correlated with PCSK9 in AAA neck. (**B**) The PPI network of PCSK9 in STRING database. The meaning of the color of the connecting lines can refer to the website (https://www.string-db.org/). (**C**) The PPI network of PCSK9 in GeneMANIA database. (**D**) TF-target and miRNA-target network of PCSK9. Green: TFs; Red: miRNA; Yellow: protein that interacted with PCSK9 from PPI network; TFs: transcription factors.

We further investigated the genes that strongly and significantly correlated with PCSK9, the top 15 genes that positively or negatively related to PCSK9 were exhibited in Fig. 8A. To investigate the potential proteins that interact with PCSK9. We used STRING and GeneMANIA datasets for the PPI network of PCSK9. As the results from STRING shown in Fig. 8B, PCSK9 interacted with LDLR, LDLRAP1, VLDLR, APOB, ANXA2, EGF, APAF1, APLP2, SORT1, and CETP. We further used GeneMANIA to explore the PPI network of PCSK9. The results illustrated that PCSK9 was related to ANXA2, LDLR, APOB, SCNN1B, ARMC6, MMP2, PSCK7, MAPK1, etc. (Fig. 8C). Afterward, 17 miRNA and 2 TFs (ETV4 and ELF3) were screened based on the based on the PPI networks. Other TFs that bind to PCSK9 were predicted by the Signaling Pathways Project database. A total of 48 TFs were acquired under the restriction of “human” in the biological sample category with FDR < 0.05. Then, the integrated network of these TFs, miRNAs, and PCSK9 was constructed as shown in Fig. 8D.

### Potential mechanisms of PCSK9 in AAA neck

**Figure 9:**
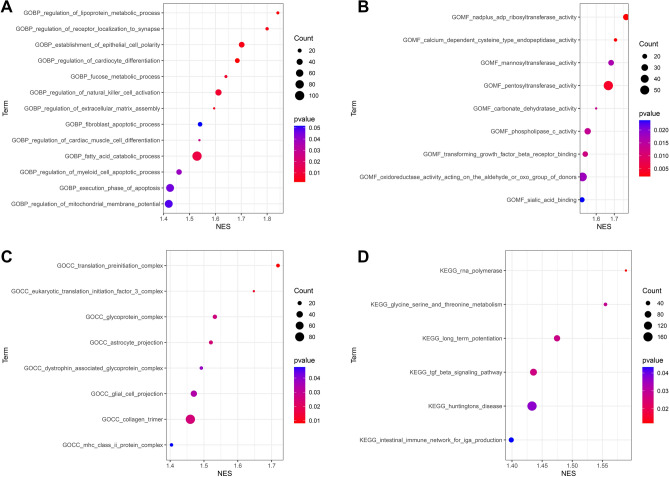
Function enrichment analysis of PCSK9 in AAA neck. (**A**) GO (biological process) analysis of PCSK9 in AAA neck. (**B**) GO (molecular function) analysis of PCSK9 in AAA neck. (**C**) GO (cell component) analysis of PCSK9 in AAA neck. (**D**) KEGG analysis of PCSK9 in AAA neck. *NES* normalized enrichment score.

To investigate the potential mechanisms of PCSK9 in AAA neck. We performed GO and KEGG analysis based on the GSEA results. The GO (biological process) analysis results indicated that PCSK9 may be involved in regulation of lipoprotein process, regulation of receptor localization to synapse, establishment of epithelial cell polarity, regulation of cardiocyte differentiation, fucose metabolic process, regulation of natural killer cell activation, regulation of extracellular matrix assembly, fibroblast apoptotic process, regulation of cardiac muscle cell differentiation, fatty acid catabolic process, regulation of myeloid cell apoptotic process, execution phase of apoptosis, and regulation of mitochondrial membrane potential (Fig. 9A). GO (molecular function) analysis of PCSK9 in AAA neck illustrated that PCSK9 may enriched in nadplus adp ribosyltransferase activity, calcium dependent cysteine type endopeptidase activity, mannosyltransferase activity, pentosyltransferase activity, carbonate dehydratase activity, phospholipase c activity, transforming growth factor beta receptor binding, oxidoreductase activity acting on the aldehyde or oxo group of donors, and sialic acid binding (Fig. 9B). GO (cell component) analysis showed that PCSK9 may be involved in translation preinitiation complex, eukaryotic translation initiation factor 3 complex, glycoprotein complex, astrocyte projection, dystrophin associated glycoprotein complex, glial cell projection, collagen trimer, and MHC class II protein complex (Fig. 9C). KEGG analysis indicated that PCSK9 may be associated with RNA polymerase, glycine serine and threonine metabolism, long term potentiation, TGF-β signaling pathway, and intestinal immune network for IGA production (Fig. 9D). These results increased the understanding of the mechanism of PCSK9 in AAA neck.

## Discussion

AAA is a common and lethal disease of the infrarenal aorta. Although increasingly identified by ultrasound screening programs worldwide, no pharmacological therapies have proven effective in limiting AAA progression or death from aneurysm rupture^29^. The AAA neck is linked to the long-term prognosis after endovascular or open AAA repair^4^. Therefore, exploring the gene profile, the mechanism of occurrence and development of AAA neck, finding suitable targets based on the mechanisms of action, and screening for and designing targeted drugs that intervene and control the progress of AAA neck have extremely significant clinical value.

In the current study, we used the data from GSE47472 (containing 14 AAA neck and 8 donor samples) in GEO dataset to investigate the DEGs and the potential role of PCSK9 in AAA neck. We firstly explored the DEGs in the neck of AAA. The DEGs contained 82 up-regulated genes and 248 down-regulated genes with the threshold at “adjusted *p* < 0.05 and |Log FC|> 1. In the DEGs, VEGFD was up-regulated in AAA neck. A previous study has demonstrated that VEGF-A and VEGFR contribute to the development in AAA through inhibition of mural angiogenesis, MMP production, myeloid cell chemotaxis, and circulating monocytes^30^. Thereafter, PCSK9 expression was evaluated in AAA neck and normal tissues. We discovered that PCSK9 was up-regulated in AAA neck than normal tissues. Our scRNAseq analysis revealed that PCSK9 was mainly expressed in in fibroblasts in AAA. Additionally, a previous study that verified the high PCSK9 intensity in aneurysm^6^. PCSK9 has been reported to directly interface with, and decrease, endogenous cell surface CD36, thus decreasing the internalization of fatty acids in adipocytes. It has also been demonstrated that PCSK9 expressed around calcification areas formed by vascular smooth muscle cells, especially those of the synthetic phenotype, was associated with the development of acute aortic dissection, suggesting that PCSK9 is associated with the loss of structural integrity of the aorta and subsequent imbalanced vasoconstriction^7^. In addition, infection of C57BL/6 mice with AAV that obtained stable expression of functionally mutated mouse PCSK9 provided a model for rapid enhancement of AngII-induced AAA^8^. These results demonstrated that PCSK9 up-regulated in AAA neck and may play a role in the development of AAA neck.

PCSK9 is regarded as a key protein in LDL cholesterol metabolism because of its critical role in LDL receptor degradation. In this study, we also verified this by the PPI network which shown the interaction between PCSK9 and LDL, LDLAP1, VLDLR, and MMP2. Initial data from investigations of PCSK9 inhibition in humans indicate that PCSK9 inhibition may be a promising novel therapeutic alternative for the treatment of dyslipidemia and related cardiovascular diseases^31,32^. Moreover, PCSK9 can undermine the recycling of MHC I to the cell surface through associating with it physically and promoting its relocation and degradation in the lysosome. PCSK9 knockout also augments the therapeutic efficacy of immune checkpoint therapy targeting the checkpoint protein PD-1. Therefore, PCSK9 inhibition is a new and hopeful approach to augment the immune checkpoint for cancer treatment^9^. However, whether there is a correlation between PCSK9 and immune checkpoints in AAA neck remains unknown. In current work, we investigated the expression of 8 immune checkpoints in AAA neck and normal tissues. We revealed that immune checkpoints PDCD1LG2 (PD-L2) was at higher expression levels in AAA neck than in normal group. However, the expression of TIGIT, PDCD1, CTLA4, and SIGLEC15 were down-regulated in the neck of AAA (Fig. 4). Moreover, PCSK9 was negatively related to CTLA4 and LAG3, and positively associated with PDCD1LG2 (PD-L2). Immune checkpoint also paly important role in the abdominal aortic aneurysm progression, for example, immune checkpoint PD-1 mediates abdominal aortic aneurysm and pseudoaneurysm progression^11^. It is well known that PD-L2 is the second ligand of PD-1^33^. Additionally, function enrichment analysis revealed that PCSK9 may be involved in several immune related biological processes in AAA neck, such as regulation of natural killer cell activation and MHC class II protein complex. PCSK9 may be involved in the development of AAA neck through immune checkpoint regulation. These results indicated that PCSK9 may be associated with immune checkpoints in AAA neck.

Ferroptosis is a newly discovered non-apoptosis-regulated form of cell death characterized by iron-dependent lipid peroxide accumulation and reactive oxygen species accumulation. The process of ferroptosis is regulated by the iron metabolism pathway, lipid metabolism pathway, glutamine catabolic pathway, and glutathione peroxidase 4 (GPX4) pathway. Among them, GPX4 is considered to be a key regulator of the occurrence of iron death and is involved in the study of the pathogenesis of various diseases^34,35^. Previous study has showed that iron level in the aorta tissue of AAA significantly elevated, iron overload induced oxidative stress and inflammatory response exacerbated AAA progression^12^. In the current study, we found that PCSK9 was negatively related to GPX4 which is a ferroptosis inhibit factor and often used as an indicator. So, we hypothesized that PCSK9 may promote ferroptosis, playing a role in the neck of AAA. Additionally, the results indicated that PCSK9 was associated with the regulation of lipoprotein process, fucose metabolic process, fibroblast apoptotic process, fatty acid catabolic process, regulation of myeloid cell apoptotic process, execution phase of apoptosis, and regulation of mitochondrial membrane potential (Fig. 9A), demonstrating that PCSK9 may regulate ferroptosis by lipid metabolism pathways. It has been illustrated that PCSK9 protein can induce ROS production^36–38^ and mitochondrial dysfunction^38^, which promote ferroptosis, demonstrating that PCSK9 may regulate ferroptosis by regulating ROS production and mitochondrial function. In addition, PCSK9 may also participate in ferroptosis by regulating the expression of TLR4^39,40^. These studies are consistent with the results of our functional enrichment analysis. Our results also demonstrated that PCSK9 was associated with other ferroptosis-related genes. However, some researchers have also found that interference with PCSK9 results in abnormal lipid metabolism, high lipid peroxidation and the ferroptosis of hepatocellular carcinoma cells^41^. This may be due to the different functions of the gene in different diseases. Therefore, the complex association between PCSK9 and ferroptosis requires more researches to explore.

In conclusion, our results demonstrated that PCSK9 was up-regulated in the neck of AAA. PCSK9 may play a role in AAA neck through the regulation of immune checkpoints and ferroptosis-related genes.

## Data Availability

The datasets generated and/or analyzed during the current study are available in the GEO repository (GSE47472: https://www.ncbi.nlm.nih.gov/geo/query/acc.cgi?acc=GSE47472; GSE164678: https://www.ncbi.nlm.nih.gov/geo/query/acc.cgi?acc=GSE164678), or from the corresponding author upon reasonable request in compliance with ethical standards.
